# Analysis of imaging in pediatric bronchopulmonary foregut malformations with literature review: case reports

**DOI:** 10.3389/fped.2024.1400124

**Published:** 2024-05-15

**Authors:** Shaohua Ji, Hong Zhang, Yan Guan, Chen Song, Meirong Han

**Affiliations:** Department of Radiology, Shanxi Children’s Hospital and Women Health Center of Shanxi, Taiyuan, Shanxi, China

**Keywords:** bronchopulmonary foregut malformation, CT, pediatrics, sequestered lung, upper gastrointestinal tract radiography

## Abstract

**Background:**

Bronchopulmonary foregut malformation (BPFM) is an uncommon condition, with few case reports documented in both national and international literature. This scarcity underscores the importance of utilizing effective imaging techniques to improve our understanding and diagnostic precision concerning this disorder.

**Case description:**

In the first case report, a neonate, born at full term and aged 15 days, presented with symptoms including dyspnea, coughing, wheezing, cyanosis, and vomiting. Initial diagnostic evaluations, which included chest radiography and upper gastrointestinal tract radiography, led to an erroneous initial diagnosis of a left-sided diaphragmatic hernia, accompanied by a suspicion of infection. In the second case report, another neonate, also born at full term but aged 5 days, exhibited symptoms such as coughing, choking, and mild vomiting. Utilizing a combination of computed tomography (CT) scans (plain, enhanced, and reconstructed), chest x-ray, and upper gastrointestinal tract radiography, the diagnosis of BPFM was accurately determined.

**Conclusion:**

Comprehensive imaging examinations play a crucial role in reducing misdiagnosis and diagnostic oversights in cases of BPFM. Given its rarity, BPFM often manifests as a sequestered lung accompanied by gastrointestinal abnormalities. Hence, the integration of CT scans with gastrointestinal tract radiography can substantially improve diagnostic precision in such cases.

## 1 Introduction

Bronchopulmonary foregut malformation (BPFM) represents a rare congenital disorder in children, with its precise origins continuing to be a subject of debate among researchers. According to relevant literature reports (1, 2), BPFM is generally believed to be a congenital developmental anomaly in the respiratory and digestive systems, which is caused by the separation of some embryonic lung tissue from the bronchial tree during embryonic lung development. It is supplied by abnormal systemic circulation and is also associated with abnormal digestive system development, such as communication with the stomach and esophagus. Some studies suggest that the development of additional accessory lung buds situated beneath the normal lung buds, which do not regress in a timely fashion and retain a common blood supply with the foregut, may contribute to the condition (3, 4). These abnormal connections may harbor bronchial epithelium or esophagogastric mucosa. The affected lung may receive its blood supply from either systemic circulation alone or from both systemic and pulmonary circulations, with venous drainage shared equally between systemic and pulmonary veins.

As early as 1968, Gerle et al. (5) were the first to propose and document BPFM. They theorized that BPFM is a cluster of anomalies involving the upper digestive tract, lungs, and arteriovenous system, stemming from a developmental interruption at a specific embryonic stage. Pulmonary sequestration is predominantly observed in the posterobasal segment of the left lower lobe and is frequently associated with diaphragmatic hernia, congenital heart disease, and other congenital abnormalities. Research suggests that the pathological basis of BPFM lies in pulmonary sequestration and esophagobronchial fistula (3, 6). Patients often exhibit recurrent respiratory symptoms and are frequently misdiagnosed with conditions such as pneumonia, congenital pulmonary airway malformation (CPAM), or congenital cystic bronchiectasis, among others (7, 8).

In recent years, the widespread use of prenatal ultrasound screening and increased emphasis on prenatal examinations have significantly contributed to a decline in congenital malformations in newborns, particularly rare anomalies. Thus, it is crucial to continuously improve awareness and diagnostic accuracy for rare diseases. In this paper, we explore a rare pediatric anomaly known as BPFM, aiming to provide more information on the diagnosis of this disorder.

## 2 Case information

### 2.1 Case 1

**Figure 1:**
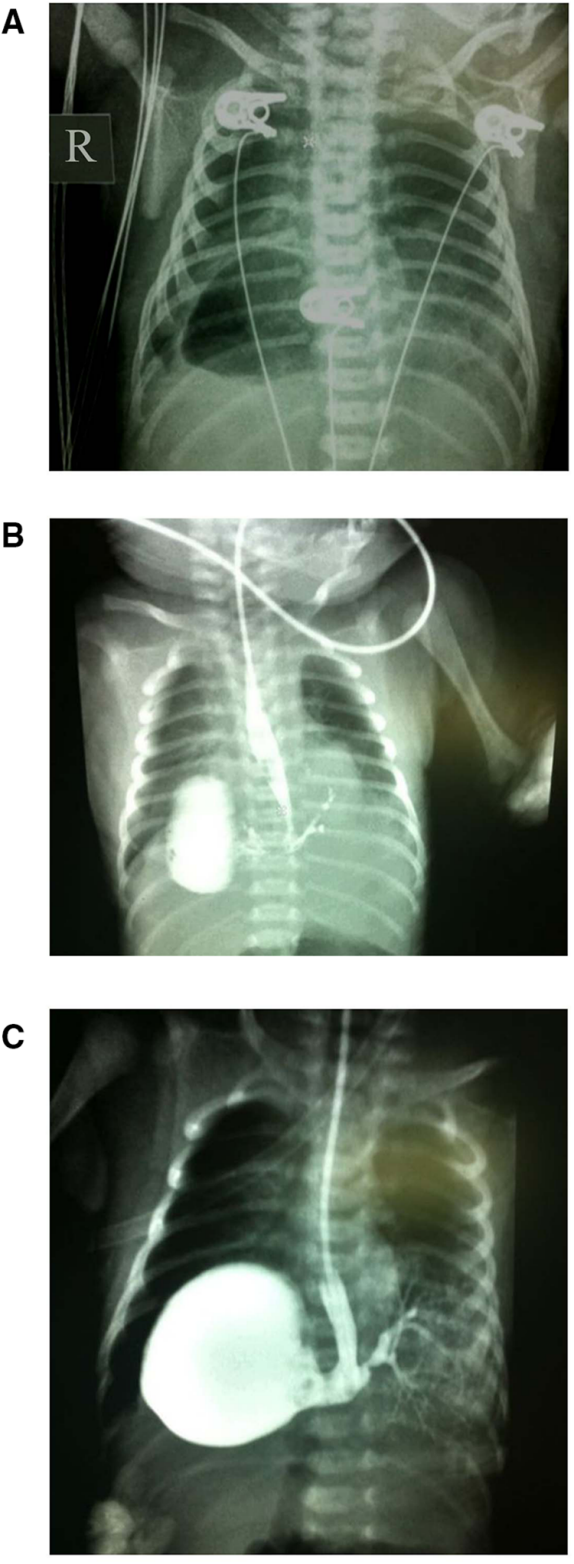
Imaging results for Case 1. (**A**) A circular clear area in the right cardiophrenic angle and a soft tissue density shadow in the lower left lung. (**B,C**) The gas-filled cavity in the right cardiophrenic angle, highlighted by a contrast agent, revealing a connection between the stomach and lower esophageal segment to the bronchus in the lesion of the lower left lung demonstrating a branching pattern.

15 day full-term newborn with symptoms such as fever, difficulty breathing, cough, wheezing, cyanosis of skin color, and vomiting. The diagnostic procedure includes chest x-ray examination and upper gastrointestinal x-ray examination. It is worth noting that the chest x-ray shows a circular transparent area observed in the right heart corner, and a slight opacity observed in the lower left lung (see Figure 1A). Initial assessments raised suspicions of a left-sided diaphragmatic hernia, possibly complicated by infection. The subsequent administration of a contrast agent through a gastric tube revealed a gas-filled cavity located at the right cardiophrenic angle. Further imaging revealed a connection between the stomach and esophagus in the right thoracic cavity and the bronchus in the lesion in the lower left lung, exhibiting a branching pattern (see Figures 1B,C).

### 2.2 Case 2

**Figure 2:**
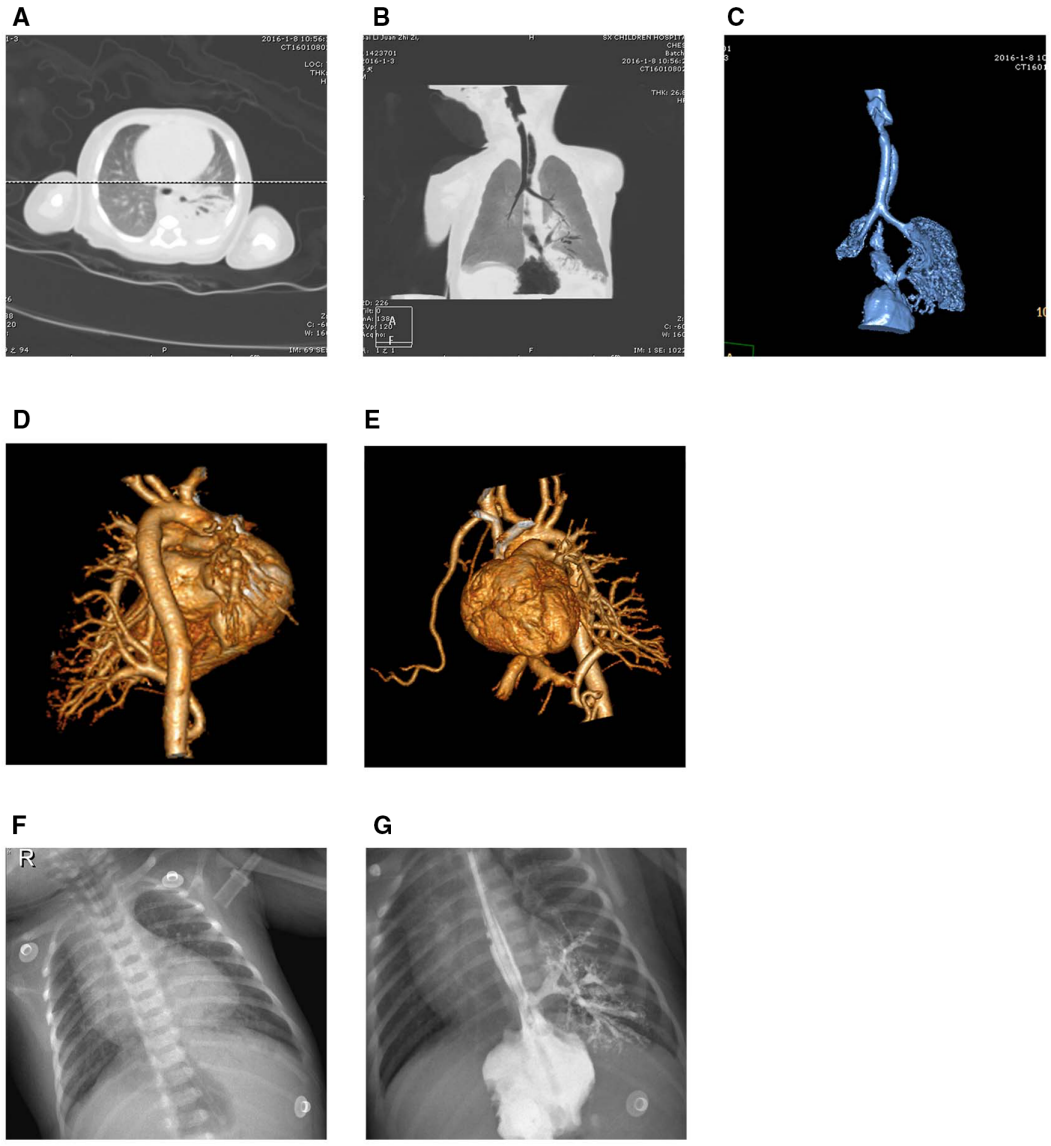
Imaging results for Case 2. (**A,B**) A soft tissue density shadow in the posterobasal segment of the lower left lung with an internal bronchial gas pattern, where the bronchial shadow within the lesion connects with the esophagus. (**C**) An abnormal passage between the sequestered lung and the esophagus. (**D,E**) The sequestered lung and its aberrant supplying artery. (**F,G**) Faint opacities in the cardiophrenic angle of the lower left lung. The contrast agent in the lower esophagus enters the lesion in the lower left lung through an abnormal passage, displaying a bronchial branching pattern.

A 5-day full-term newborn with symptoms of cough, suffocation, vomiting, and cyanosis around the mouth, without fever. Diagnostic imaging included CT scans, chest x-ray, and upper gastrointestinal tract radiography. The chest x-ray displayed coarsened and blurred pulmonary textures with patchy areas of increased density observed behind the cardiac silhouette in the left lung (see Figure 2F). This suggested a potential exudative lesion in the lower left lung. Introduction of a contrast agent into the lower esophagus revealed its entry into lesion in the lower left lung through an abnormal passage, revealing a bronchial branching pattern in the imaging (see Figure 2G). The plain CT scan depicted a soft tissue density shadow in the posterobasal segment of the lower left lung with an internal bronchial gas pattern (see Figure 2A). Minimum intensity projection (minIP) imaging illustrated the bronchial shadow within the lesion connecting to the esophagus (see Figure 2B). Volume rendering (VR) imaging revealed a tubular shadow extending downward connecting with the esophagus (see Figure 2C). The sequestered lung was vascularized by a branch from the abdominal aorta and drained into the lower left pulmonary vein (see Figures 2D,E).

## 3 Discussion

Recent literature surveys have highlighted the ongoing debates surrounding the definition of BPFM. Certain studies have suggested that BPFM represents a spectrum of diverse yet related diseases, involving the bronchial tree, lung parenchyma, gastrointestinal tract, diaphragm, and vascular system (9, 10). This proposed spectrum encompasses malformations arising from both endodermal and mesodermal origins, such as congenital pulmonary cystic diseases like lobar emphysema, bronchogenic cyst, CPAM, and sequestered lung, among others. Additionally, it includes conditions such as pulmonary agenesis, pulmonary aplasia or hypoplasia, and various congenital anomalies of the digestive system including tracheoesophageal fistula, esophageal atresia, and esophageal duplication, among others (11).

Srikanth et al. (12) categorized BPFM into four types: Type I involves esophageal atresia with the atretic distal segment forming a fistula with the sequestered lung tissue, further subdivided into IA (affecting one side of the lung) and IB (impacting a single lobe or segment of the lung); Type II is characterized by the absence of one lung with sequestered lung tissue in the thoracic cavity originating from the lower segment of the esophagus; Type III includes a sequestered lobe or lung segment communicating with the esophagus or stomach; Type IV entails a portion of the normal bronchial system communicating with the esophagus. Literature reports indicate that Type III is the most frequently encountered (13, 14). The two cases presented in this study fall under Type III, aligning with the trends observed in the literature.

In analyzing the two cases in this study, the misdiagnosis and overlooked diagnosis in Case 1 primarily stemmed from the limited diagnostic capability of conventional chest x-rays in identifying the imaging features of a sequestered lung. These x-rays inadequately displayed the abnormal vascularization of the sequestered lung and its aberrant communication with the digestive tract. Although upper gastrointestinal tract radiography revealed the abnormal passage between the digestive tract and the sequestered lung, it failed to showcase the vascular supply to the lesion, thus providing an incomplete assessment. Furthermore, the clinical symptoms of the neonate (predominantly respiratory infection signs and vomiting in conjunction with the chest x-ray findings) led to a misdiagnosis of left-sided diaphragmatic hernia with a suspected concurrent lung infection. The key reasons for this misdiagnosis and oversight were inadequate clinical assessment and over-reliance on a single imaging modality.

In Case 2, the utilization of chest CT scans, both plain and enhanced, not only delineated the extent of the lesion and its relationship with the airways, but also illustrated the vascular supply to the lesion. This comprehensive strategy, incorporating chest x-rays and gastrointestinal tract radiography, markedly enhanced diagnostic precision for this condition.

BPFM is a rare and complex abnormality characterized by an abnormal passage between the gastrointestinal tract and a sequestered lung. Its imaging hallmarks encompass: (1) signs of a sequestered lung, typically appearing as a mass-like or cystic soft tissue density shadow within the lung, most commonly observed in the posterobasal segment of the left lower lobe; (2) an anomalous connection between the lung lesion and the digestive tract.

In differential diagnosis, due to the rarity of BPFM as a congenital anomaly, it is prone to misdiagnosis and oversight, necessitating its distinction from other more prevalent conditions such as CPAM, lobar pneumonia, and congenital cystic bronchiectasis. CPAM is typically evident on CT scans as multiple or single variably-sized thin-walled cysts or cystic-solid lesions with differing degrees of mass effect (15). Lobar pneumonia radiologically presents as exudation and consolidation of various shapes and sizes, with characteristic CT findings of uniformly dense consolidation shadows in a lung lobe or segment, including air bronchograms, and resolution following anti-infection treatment (16). Congenital cystic bronchiectasis generally appears on CT scans as multiple circular air-filled cysts in a bead-like arrangement, sometimes with air-fluid levels inside the cysts (17).

Recent advancements in low-dose CT technology have been instrumental in providing diagnostically adequate images while concurrently mitigating the deleterious effects of radiation exposure, particularly in the pediatric population, including infants (18). The study of low-dose CT examination is crucial for the diagnosis of pediatric diseases and is also the core of the development of pediatric CT. By optimizing scanning parameters and using advanced image reconstruction techniques, we ensure image quality while shortening children's examination time, thereby reducing CT radiation dose. Research has shown that the radiation dose of low-dose CT examination in children is only about 1/5 of that of ordinary CT, especially with the superiority of three-dimensional reconstruction technology in observing special structures such as airways and blood vessels (19). Therefore, adopting such technologies along with an appropriate sedation protocol is essential. The combined application of enhanced CT and gastrointestinal tract radiography has proven to be an effective method for accurate diagnosis. Moreover, it plays a vital role in guiding surgical treatment choices and in prognostic evaluation.

## 4 Conclusion

In conclusion, BPFM represents a rare and complex anomaly involving the digestive, respiratory, and vascular systems. Precise diagnosis of each constituent malformation necessitates the utilization of advanced imaging techniques. However, given the challenges posed by the age group (newborns) and their limited ability to cooperate during examinations, thoughtful consideration should be given to incorporating sedation or immobilization techniques. Additionally, the potential radiation impact of CT scans on infants warrants careful assessment in clinical practice. Low-dose CT examination not only promotes effective imaging and enhances image fidelity but also significantly reduces radiation exposure in infants, which can be widely promoted in clinical practice.

## Data Availability

The original contributions presented in the study are included in the article/Supplementary Material, further inquiries can be directed to the corresponding author.
